# Persistence of the historical lineage I of West Africa against the ongoing spread of the Asian lineage of peste des petits ruminants virus

**DOI:** 10.1111/tbed.14066

**Published:** 2021-03-23

**Authors:** Kadidia Tounkara, Olivier Kwiatek, Cheick Abou Kounta Sidibe, Amadou Sery, Marthin Dakouo, Habib Salami, Modou Moustapha Lo, Aminata Ba, Mariame Diop, Mamadou Niang, Geneviève Libeau, Arnaud Bataille

**Affiliations:** ^1^ CIRAD UMR ASTRE Montpellier France; ^2^ ASTRE Univ Montpellier CIRAD INRA Montpellier France; ^3^ Laboratoire Central Vétérinaire (LCV) Bamako Mali; ^4^ Institut Sénégalais de Recherches Agricoles Laboratoire National d'Elevage et de Recherches Vétérinaires (LNERV) Dakar‐Hann Sénégal

**Keywords:** disease control, molecular epidemiology, Morbillivirus, phylogeny, small ruminant, transboundary

## Abstract

Peste des petits ruminants (PPR) is a highly contagious disease of small ruminants. The causal agent, PPR virus (PPRV), is classified into four genetically distinct lineages. Lineage IV, originally from Asia, has shown a unique capacity to spread across Asia, the Middle East and Africa. Recent studies have reported its presence in two West African countries: Nigeria and Niger. Animals are frequently exchanged between Mali and Niger, which could allow the virus to enter and progress in Mali and to other West African countries. Here, PPRV samples were collected from sick goats between 2014 and 2017 in both Mali and in Senegal, on the border with Mali. Partial PPRV nucleoprotein gene was sequenced to identify the genetic lineage of the strains. Our results showed that lineage IV was present in south‐eastern Mali in 2017. This is currently the furthest West the lineage has been detected in West Africa. Surprisingly, we identified the persistence at least until 2014 of the supposedly extinct lineage I in two regions of Mali, Segou and Sikasso. Most PPRV sequences obtained in this study belonged to lineage II, which is dominant in West Africa. Phylogenetic analyses showed a close relationship between sequences obtained at the border between Senegal and Mali, supporting the hypothesis of an important movement of the virus between the two countries. Understanding the movement of animals between these countries, where the livestock trade is not fully controlled, is very important in the design of efficient control strategies to combat this devastating disease.

## 1 INTRODUCTION

Peste des petits ruminants (PPR) is a highly acute contagious transboundary infectious disease of small ruminants, primarily sheep and goats. It is one of the most economically important animal diseases in areas that rely on small ruminants for their livelihoods. The main clinical signs of PPR are the rapid rise in body temperature, the appearance of nasal and ocular secretions which become mucopurulent, necrotizing stomatitis, profuse, haemorrhagic diarrhoea in cases of parasitism and death. Signs of pneumonia (cough, dyspnoea), abortions that can be observed among pregnant animals, are important clinical aspects of PPR; because of its high contagiousness, high morbidity and mortality, PPR is a World organization for Animal Health (OIE) notifiable disease and since 2015 targeted for global eradication by 2030 (FAO/OIE, 2015).

Peste des petits ruminants is endemic in large parts of Africa, the Middle East and Asia (Libeau et al., 2014). PPR is caused by peste des petits ruminants virus (PPRV) a member of the *Morbillivirus* genus in the *Paramyxoviridae* family [species: *Small ruminant morbillivirus*; (Amarasinghe et al., 2017)]. PPRV is a negative sense single‐stranded ribonucleic acid (RNA) virus with an envelope varying in shape and size, ranging from 170 nm to 500 nm (Bourdin & Laurent‐Vautier, 1967; Durojaiye et al., 1985). The PPRV genome has a length between 15,948 pb and 15,954 pb and encodes six structural proteins: nucleoprotein (N), phosphoprotein (P), matrix protein (M), fusion protein (F), haemagglutinin protein (H) and a viral RNA‐dependent polymerase (L) and two non‐structural proteins C and V (Parida et al. 2015).

The PPR virus strains are divided into four genetic lineages (I, II, III and IV) based on N and F genes partial sequences (Kwiatek et al., 2007; Shaila et al., 1989). All PPRV lineages are circulating in the African continent: I and II in West Africa, III in East Africa and IV in Central, East, North and West Africa (Baazizi et al., 2017; Banyard et al., 2010; Dundon et al., 2020; Kwiatek et al., 2011; Muniraju et al., 2014). The lineages II and IV have the widest distribution, appearing to have replaced lineage I and III in West and East Africa, respectively (Dundon et al., 2020). Notably, the last confirmed presence of the lineage I in West Africa was in Senegal in 1994 and in Niger in 2001 (Tounkara et al., 2018). Lineage II PPR strains are now widespread across West Africa, probably linked with extensive transboundary animal movements (Tounkara et al., 2018). Strains of lineage IV have also started to emerge in West Africa in recent years, with a wide diversity of strains already described in Nigeria (Mantip et al., 2016; Woma et al., 2016), and a first description in Niger in 2013 (Souley et al., 2019; Tounkara et al., 2018). The lineage may have already spread further westward with the commercial movement of infected animals, but data are currently lacking to evaluate this risk.

The objective of this study is to undertake molecular epidemiology analysis (phylogenetic and phylogeographic) of PPR virus strains identified in most of the administrative regions of Mali between 2014 and 2017. Mali is a crossroads for the passage of small ruminants from West African border countries. Recurrently, there are many exchanges of animals between Mali and Niger, which could allow the virus to enter and let lineage IV continue to progress across Mali. From Mali, it could spread to other West African countries. We explored this hypothesis by carrying out sampling at major crossing points at the border between Mali and Senegal, a major endpoint for livestock in West Africa.

## 2 MATERIALS AND METHODS

### 2.1 Ethical approval, sampling and RNA extraction

**FIGURE 1:**
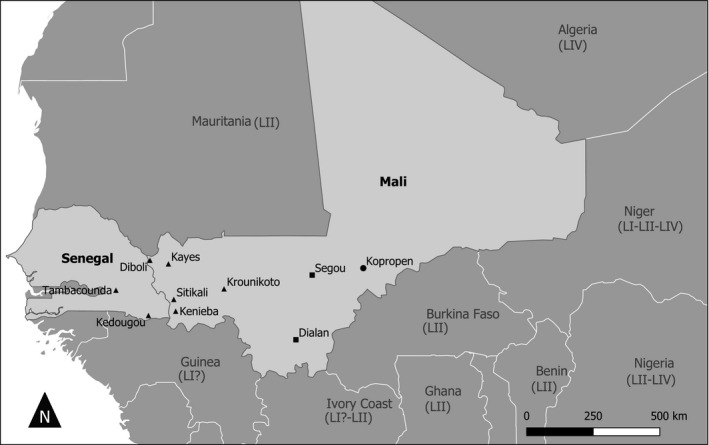
Map of Mali and Senegal showing sampling location. Rectangles indicate the samples belonging to the Lineage I. Dots represent location of samples belonging to the lineage IV and the triangles those belonging to the lineage II

Samples were collected between 2014 and 2017 from domestic small ruminants (goat) showing clinical signs suggesting PPR infection by Mali's Central Veterinary Laboratory (LCV). These were collected in different towns in the regions of Kayes, Segou, Sikasso and Mopti (Figure 1). In addition, samples were collected in 2017 by the Institut Sénégalais de Recherche Agricole (ISRA) in two villages in Senegal (Kedougou and Tambacounda) on the border with Mali. The field sampling was carried out by the national veterinary services staff following national legislation, which does not require ethical approval. Nevertheless, the tissues for this study were collected respecting animal welfare. The study focussed on animals in outdoor environment where PPR is endemic. Animals showing symptoms corresponding to PPR infection were restrained to obtain ocular and nasal swabs. The tissue samples (lung, lymph nodes and/or spleen) were collected from animals, who died from infection or humanely killed after developing acute PPR symptoms, including mucopurulent nasal/ocular discharges, fever, diarrhoea, respiratory distress and loss of weight. Samples were stored at 4°C during their transfer to the central veterinary laboratories and sent to CIRAD Montpellier France for processing in a Biosafety Level 3 containment Laboratory.

In the laboratory, the tissue samples were cut into small pieces and put in 15‐ml tube then vortexed with 3 ml of Minimum essential Medium (MEM) (Invitrogen) and 2 mm glass beads. The swabs were processed in 1 ml of MEM and vortexed. All the sample suspensions were then centrifuged for 3 min at 1,000 g and the supernatant collected. RNA extraction was carried out using the NucleoSpin RNA virus extraction kit (Macherey‐Nagel) and according to the instructions of the manufacturer. A 351 base pair (bp) fragment of the PPRV N gene was amplified by RT‐PCR carried out using the qScript XLT One‐Step RT‐PCR kit (Quantabio, VWR) using the protocol described Tounkara et al., (2018).

### 2.2 Sequencing and phylogenetic analysis

Positive PCR products were cleaned up and sequenced in both forward and reverse directions by the Genewiz sequencing platform (United Kingdom) and the sequences submitted to GenBank. Sequence assemblage and trimming were performed using Geneious v. 8.1.6 (final size of sequence = 255 bp). Clean sequences were aligned using MEGA 7 with 53 other sequences available in GenBank, including representatives of the four genetic lineages identified in Africa (see Appendix S1). Phylogenetic relationship between sequences was inferred with a maximum likelihood method as implemented in MEGA 7, with node supports calculated by bootstrap analyses (1,000 replicates).

### 2.3 Isolation of the virus

A total of 12 samples were selected based on RT‐PCR results to attempt virus isolation with CHS‐20 cells with 1% antibiotics and 10% foetal bovine serum (Invitrogen) (Adombi et al., 2011). One negative and one positive (PPRV isolate from Ivory Coast) controls were included in the virus isolation attempts. The cells were checked daily for signs of cellular cytopathic effects (CPE) under a microscope. If no CPE were observed after 1 week, cells were trypsinized and passaged in fresh medium with only 5% of foetal bovine serum. If no CPE was observed after 2 weeks following the blind passage, the virus isolation was considered negative. Cells with CPE were stored in the freezer at −80°C.

## 3 RESULTS AND DISCUSSION

Swabs and/or tissue samples were collected from a total of 318 and 43 goats in Mali and Senegal, respectively (Table 1). RT‐PCR results were positive for 53 samples from Mali and 13 samples from Senegal. The positive samples came from all the sites visited, with the exception of Kayes and Seroume in the Kayes region (Figure 1). PPRV was successfully isolated from one nasal swab collected in Samako (Kayes region) and one lung collected in Bamako. A partial N gene sequence was obtained from 30 and 12 positive samples collected in Mali and Senegal, respectively (Table 1, Table S1). Many sequences obtained were identical, with a total of 15 unique partial N gene sequences eventually identified (GenBank accession numbers MW281010, MW281012–MW281024, MW281026). All the sequences obtained during sequencing of the partial N gene were used for phylogenetic analysis.

**TABLE 1:** Samples collected in Mali and Senegal

Region	Site	*N*	Type of sample collected	Npos	Year	GenBank
Mali						
Kayes	Kenieba	110	Swab	9 (4)	2016	MW281018, MW281019
	Sitakili	55	Swab	10 (3)	2016	MW281017, MW281021
	Kayes	57	Swab	0 (0)	2016	
	Diboli	50	Swab	5 (0)	2016	
	Krounikoto	10	Swab	8 (5)	2017	MW281020
	Seroume	6	Swab	0 (0)	2017	
Sikasso	Dialan	16	Swab, Tissue	7 (4)	2015	MW281010
Segou	Segou	5	Tissue	5 (5)	2014	MW281012‐MW281016
Mopti	Kopropen	9	Swab	9 (9)	2017	MW281016
Senegal						
Kedougou	Kedougou	11	Swab	6 (6)	2017	MW281022, MW281023
Tambacounda	Tambacounda	32	Swab	7 (6)	2017	MW281020, MW281024
						MW281026
Total		361		66 (58)		15

Note

*N*, number of samples collected; only goats were sampled in this study; Npos, number of samples tested positive for PPR genetic material using RT‐PCR and number successfully sequenced between brackets; Year, year of sampling; GenBank, GenBank accession number.

**FIGURE 2:**
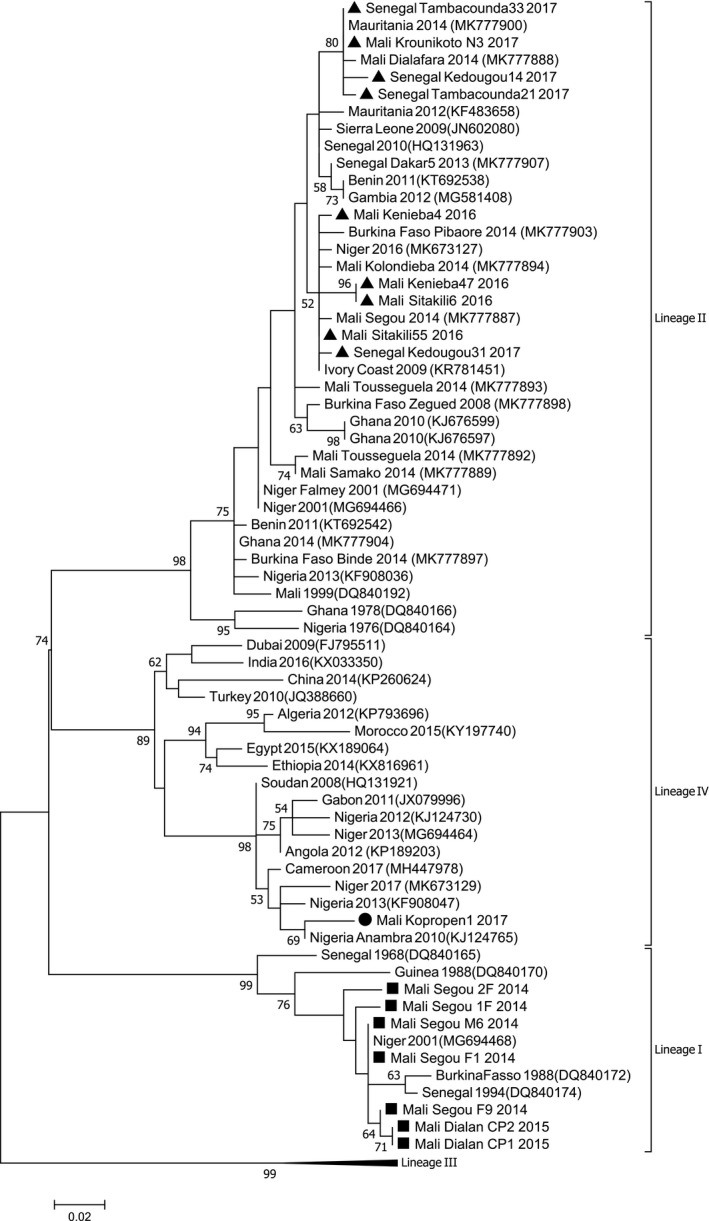
Peste des petits ruminants N gene analysis. Phylogenetic tree is constructed by using a maximum likelihood method and showing the relationship based on N gene sequences of peste des petits ruminants virus (PPRV) samples. Samples collected in this study are indicated by black dots. The numbers at the nodes are bootstrap values obtained from 1,000 replicates

Phylogenetic analyses showed that the PPRV sequences obtained during this study belonged to lineages I, II or IV (Figures 1 and 2). Seven samples collected in Mali in Segou (2014) and Dialan (2015) are clustered within lineage I. This lineage was considered to have disappeared, as it had not been described in any country since 1994. A recent article identified its presence in Niger in 2001 (Tounkara et al., 2019). Two sequences from Segou are identical to a sequence obtained in Niger in 2001. Sequences from Dialan formed a cluster with one sequence from Segou separated from other sequences from Segou (node support = 64%; Figure 2). These results suggest that lineage I continues to circulate in this region of Mali, possibly because the small ruminant trade is not important between these villages and the rest of Mali, where other strains of supposedly more dominant lineages (II and IV) are circulating. This conclusion is supported by the information gathered in situ from inhabitants. The difficulty of obtaining samples from these regions in Mali and in the neighbouring countries of Burkina Faso and Niger certainly constitutes a bias in the evaluation of the extent and diversity of lineage I strains.

One sequence obtained from a sample collected in 2017 in Kopropin (Mopti region) was placed within lineage IV (Figure 2). This site is close to the main point of entry from Niger. Currently, this is the most western detection of the Asian lineage in West Africa and follows the detection of lineage IV in Niger and Nigeria in 2013 (Mantip et al., 2016; Souley et al., 2019; Tounkara et al., 2018; Woma et al., 2016). The sequence was placed within a cluster with 69% node support that included PPRV strains from Nigeria (Figure 2), presented in earlier studies as belonging to a sub‐cluster named IV_NigB (Mantip et al., 2016; Woma et al., 2016). It is likely that lineage IV entered Mali through the important livestock trade taking place between Mali, Niger and Nigeria, and especially between Mopti (Mali), Nigeria and Niger (which are very close geographically) (Apolloni et al., 2019; Souley et al., 2019). According to the information gathered from veterinarians based in Mopti, some of the sampled animals were imported from neighbouring countries. An earlier study failed to detect lineage IV in samples collected in Burkina Faso in 2014 (Tounkara et al., 2019). Further sampling should be performed to clarify whether lineage IV has already spread to Burkina Faso, Benin, Ghana, and within other West African countries between Nigeria and Mali.

Most samples obtained belonged to lineage II, the most abundant lineage in the region (Souley et al., 2019; Tounkara et al., 2019). All PPRV sequences obtained from Senegal (Kolondieba and Kedougou) and from five samples collected in Krounikoto in the Kayes region in Mali belonged to this lineage (Figures 1 and 2). Some sequences from Senegal and Mali were identical. The phylogenetic analysis showed that sequences from Sitakili and Kenieba (border region of Kayes in Mali) and one sequence from the border region of Kedougou in Senegal formed a cluster with low node support (52%) with other sequences obtained from a previous study in Mali in 2014 and sequences from other West African countries (Figure 2). The close relationship between sequences obtained at the border between Senegal and Mali supports the hypothesis of an important movement of the virus between the two countries. Other sequences obtained across Senegal and Mali, and in Mauritania, are grouped in another cluster with good node support (80%). These results suggest that transboundary movements could take place through long‐distance animal transportation.

The great diversity of strains found in Mali provides further support for the extensive circulation of the virus in the region (Tounkara et al., 2019). The close trading relationship between West African countries appears to be the main route of PPRV spread. Notably, Mali exports hundreds of thousands of animals during Muslim festivals (Aid El Fitr and sheep festivals) to several neighbouring countries including Senegal, Niger, Benin and Nigeria. Our results suggest that the risk of introducing PPRV lineage IV in Senegal is now quite high. Continuous sampling and genotyping of small ruminants showing clinical signs suggesting PPR infection at the border between Mali and Senegal should be conducted. Such a strategy could provide the opportunity to follow in real time the spread of this lineage in the well‐described trade system (Jahel et al., 2020), and possibly to better understand the reason for its capacity to spread so successfully in PPR endemic countries. These results need to be confirmed by phylogenetic analysis of the complete genome of these strains. Phylogenomic analysis would also provide more information regarding the evolution of PPRV, notably lineages II and IV in West Africa (Adombi et al., 2016; Dundon et al., 2020).

In conclusion, our study suggests that three different PPRV lineages are currently co‐circulating in Mali. Despite the dominance of lineages II and IV, lineage I may continue to circulate in isolated regions. We detected the presence of PPRV lineage IV in Mali, and it is possible that this lineage is now spreading across the country and in other West African countries through animal trade and transhumance. Understanding the animal movements in the African States belonging to ECOWAS where free movements for the livestock trade are allowed through regional regulations is very important for the PPR eradication programme. This would improve our capacity to design efficient control strategies against this devastating disease.

## CONFLICT OF INTEREST

The authors declare that there is no conflict of interest.

## Supporting information

App S1

## Data Availability

The data that support the findings of this study are openly available in GenBank at https://www.ncbi.nlm.nih.gov/, accession numbers MW281010–MW281026.
