# Supplementary material for: Persistence of the historical lineage I of West Africa against the ongoing spread of the Asian lineage of peste des petits ruminants virus
Source: Transbound Emerg Dis. 2021 Mar 23;68(6):3107–13. doi: 10.1111/tbed.14066 (PMC9292845; doi:10.1111/tbed.14066)
Supplement: Supplementary file 1 — App S1 [file TBED-68-3107-s001.docx]

**Supplementary Material**

**Table S1.** Detailed list of partial peste des petits ruminants virus N gene sequenced from samples collected in Mali and Senegal

| Sample name | Country / Region | Year | Lineage | Genbank number |
| --- | --- | --- | --- | --- |
| MALI_DIALAN_CP1_2015 | Mali/Sikasso | 2015 | I | MW281010 |
| MALI_DIALAN_CP2_2015 | Mali/Sikasso | 2015 | I | MW281010 |
| MALI_ DIALAN_CP3_2015 | Mali/Sikasso | 2015 | I | MW281010 |
| MALI_DIALAN_CP4_2015 | Mali/Sikasso | 2014 | I | MW281010 |
| MALI_KENIEBA34 _2016 | Mali/Kayes | 2016 | II | MW281018 |
| MALI_KENIEBA39 _2016 | Mali/Kayes | 2016 | II | MW281019 |
| MALI_KENIEBA43_2016 | Mali/Kayes | 2016 | II | MW281019 |
| MALI_KENIEBA47_2016 | Mali/Kayes | 2016 | II | MW281019 |
| MALI_KOPROPEN3_2017 | Mali/Mopti | 2017 | IV | MW281026 |
| MALI_KOPROPEN5_2017 | Mali/Mopti | 2017 | IV | MW281026 |
| MALI_KOPROPEN7_2017 | Mali/Mopti | 2017 | IV | MW281026 |
| MALI_KOPROPEN1_2017 | Mali/Mopti | 2017 | IV | MW281026 |
| MALI_KOPROPEN8_2017 | Mali/Mopti | 2017 | IV | MW281026 |
| MALI_KOPROPEN9_2017 | Mali/Mopti | 2017 | IV | MW281026 |
| MALI_KOPROPEN10_2017 | Mali/Mopti | 2017 | IV | MW281026 |
| MALI_KOPROPEN14_2017 | Mali/Mopti | 2017 | IV | MW281026 |
| MALI_KOPROPEN15_2017 | Mali/Mopti | 2017 | IV | MW281026 |
| MALI_KROUNIKOTO_N1_2017 | Mali/Kayes | 2017 | II | MW281020 |
| MALI_KROUNIKOTO_N2_2017 | Mali/Kayes | 2017 | II | MW281020 |
| MALI_KROUNIKOTO_N3_ 2017 | Mali/Kayes | 2017 | II | MW281020 |
| MALI_KROUNIKOTO_N4_2017 | Mali/Kayes | 2017 | II | MW281020 |
| MALI_KROUNIKOTO_N5_ 2017 | Mali/Kayes | 2017 | II | MW281020 |
| MALI_1F_SEGOU_2014 | Mali/Ségou | 2014 | I | MW281016 |
| MALI_2F_SEGOU_2014 | Mali/Ségou | 2014 | I | MW281012 |
| MALI_F9_SEGOU_2014 | Mali/Ségou | 2014 | I | MW281013 |
| MALI_F1_NAS_SEGOU_2014 | Mali/Ségou | 2014 | I | MW281014 |
| MALI_M6_SEGOU_2014 | Mali/Ségou | 2014 | I | MW281015 |
| MALI_SITAKILI6_2016 | Mali/Sikasso | 2016 | II | MW281021 |
| MALI_SITAKILI55_2016 | Mali/Sikasso | 2016 | II | MW281017 |
| MALI_SITAKILI56_2016 | Mali/Sikasso | 2014 | II | MW281017 |
| SENEGAL_KEDOUGOU14_2017 | Sénégal/Kédougou | 2017 | II | MW281022 |
| SENEGAL_ KEDOUGOU31_2017 | Sénégal/Kédougou | 2017 | II | MW281023 |
| SENEGAL_ KEDOUGOU15_2017 | Sénégal/Kédougou | 2017 | II | MW281022 |
| SENEGAL_ KEDOUGOU33_2017 | Sénégal/Kédougou | 2017 | II | MW281022 |
| SENEGAL_ KEDOUGOU16_2017 | Sénégal/Kédougou | 2017 | II | MW281022 |
| SENEGAL_ KEDOUGOU34_2017 | Sénégal/Kédougou | 2017 | II | MW281023 |
| SENEGAL_TAMBACOUNDA88_2017 | Sénégal/Tambacounda | 2017 | II | MW281025 |
| SENEGAL_ TAMBACOUNDA21_2017 | Sénégal/Tambacounda | 2017 | II | MW281024 |
| SENEGAL_ TAMBACOUNDA33_2017 | Sénégal/Tambacounda | 2017 | II | MW281020 |
| SENEGAL_ TAMBACOUNDA47_2017 | Sénégal/Tambacounda | 2017 | II | MW281020 |
| SENEGAL_ TAMBACOUNDA75_2017 | Sénégal/Tambacounda | 2017 | II | MW281020 |
| SENEGAL_ TAMBACOUNDA96_2017 | Sénégal/Tambacounda | 2017 | II | MW281020 |

Country/region, country and region of sampling; year, year of sampling; Lineage, PPRV genetic lineage based on phylogenetic analysis; Genbank number; Genbank accession number for the partial PPRV N gene.

**SM1.** Alignment of partial PPR N gene sequences used for phylogenetic analyses.

>Mali Dialafara 2014 (MK777888)

CCTTCCTCCAGCACAAAATAGGTGAGGGAGAGTCGCCTACACCAGCGACCAAAGAAGAAGTCAAAGCTGCGATCCCAAACGGATCTGAAGGAAGAGACACAAAACGAACACGCCCAGGGAAGCCCAGAGGAGAAACTCCCGGGCAACTGCTTCTGGAGATCATGCCAGAGGATGGAGTCTCGCGAGAGTCTGGTCAAAGCCCTCGTGAGGCTCAAAGATCGGCTGAGGCACTTTTCAGGCTGCAGGCCATGGCCA

>Mali Segou 2014 (MK777887)

CCTTCCTCCAGCACAAAATAGGTGAGGGAGAGTCGCCTACACCAGCAACCAAAGAAGAAGTCAAAGCTGCGATCCCAAACGAATCTGAAGGAAGGGACATAAAGCGAACACGCCCAGGGAAGCCCAGAGGAGAAACTCCCGGGCAACTGCTTCTGGAGATCATGCCAGAGGATGGAGTCTCGCGAGAGTCTGGTCAAAACCCTCGTGAGGCTCAAAGATCGGCTGAGGCACTCTTCAGGCTGCAGGCCATGGCCA

>Mali Tousseguela 2014 (MK777893)

CCTTCCTCCAGCACAAAATAGGTGAGGGAGAGTCGCCTACACCAGCGACCAAAGAAGGAGTCAAAGCTGCGATCCCAAACGGATCTGAAGGAAGGGACATAAAGCGAACACGCCCAGGGAAGCCCAGAGGAGAAACTCCCGGGCAACTGCTTCTGGAGATCATGCCAGAGGATGAATTCTCGCGAGAGTCTGGTCAAAACCCTCGTGAGGCTCAAAGATCGGCTGAGGCACTCTTCAGGCTGCAGGCCATGGCCA

>Mali Kolondieba 2014 (MK777894)

CCTTCCTCCAGCACAAAATAGGTGAGGGAGAGTCGCCTACACCAGCAACCAAAGAAGAAGTCAGAGCTGCGATCCCAAACGGATCTGAAGGAAGGGACATAAAGCGAACACGCCCAGGGAAGCCCAGAGGAGAAACTCCCGGGCAACTGCTTCTGGAGATCATGCCAGAGGATGGAGTCTCGCGAGAGTCTGGTCAAAACCCTCGTGAGGCTCAAAGATCGGCTGAGGCACTCTTCAGGCTGCAGGCCATGGCCA

>Mali Kenieba47 2016 (MW281019)

CCTTCCTCCAGCACAAAATAGGTGAGGGAGAGTCGCCTATACCAGCAACCAAAGAAGAAGTCAAAGCTGCGATCCCAAACGGATCTGAAGGAAGGGACATAAAGCGAACACTCCCAGGGAAGCCCAGAGGAGAAACTCCCGGGCAACTGCTTCTGGAGATCATGCCAGAGGATGGAGTCTTGCGAGAGTCTGGTCAAAACCCTCGTGAGGCTCAAAGATCGGCTGAGGCACTCTTCAGGCTGCAGGCCATGGCCA

>Mali Kenieba4 2016 (MW281018)

CCTTCCTCCAGCACAAAATAGGTGAGGGAGAGTCGCCTACACCAGCAACCAAAGAAGAAGTCAAAGCTGCGATCCCAAACGGATCTGAAGGAAGGGACATAAAGCGAACACGCCCAGGGAAGCCCAGAGGAGAAACTCCCGGGCAACTGCTTCTGGAGATCATGCCAGAGGATGGGGTCTCGCGAGAGTCTGGTCAAAACCCTCGTGAGGCTCAAAGATCGGCTGAGGCACTCTTCAGGCTGCAGGCCATGGCCA

>Mali Kopropen1 2017 (MW281026)

CCTTCCTCCAGCACAAACCGGGAGAGGGAGAGTCGCCCACACCAGCGACTAGAGAAGGGGTCAAAGCTGCGATCCCAAACGGATCTGAAGAGAGGGAAAGAAAGCAAACACGTTCAGGAAGGCCCAGAGGGGAGACCCCCGGCCAACCGCTCCTGGAAATCACGCCAGAGGACGAGGTCCCGCGAGAGTCTGGTCAAAACCCTCGTGAGGCTCAAAGATCGGCCGAGGCACTCTTCAGGCTGCAGGCCATGGCCA

>Mali Krounikoto N3 2017 (MW281020)

CCTTCCTCCAGCACAAAATAGGTGAGGGAGAGTCGCCTACACCAGCGACCAAAGAAGAAGTCAAAGCTGCGATCCCAAACGGATCTGAAGGAAGAGACACAAAACGAACACGCCCAGGGAAGCCCAGAGGAGAAACTCCCGGGCAACTGCTTCTGGAGATCATGCCAGAGGATGGAGTCTCGCGAGAGTCTGGTCAAAACCCTCGTGAGGCTCAAAGATCGGCTGAGGCACTTTTCAGGCTGCAGGCCATGGCCA

>Mali Sitakili6 2016 (MW281021)

CCTTCCTCCAGCACAAAATAGGTGAGGGAGAGTCGCCTATACCAGCAACCAAAGAAGAAGTCAAAGCTGCGATCCCAAACGGATCTGAAGGAAGGGACATAAAGCGAACACTCCCAGGGAAGCCCAGAGGAGAAACTCCCGGGCAACTGCTTCTGGAGATCATGCCAGAGGATGGAGTCTTGCGAGAGTCTGGTCAAAACCCTCGTGAGGCTCAAAGATCGGCTGAGGCACTCTTCAGGCTGCAGGCCATGGCCA

>Mali Sitakili55 2016 (MW281017)

CCTTCCTCCAGCACAAAATAGGTGAGGGAGAGTCGCCTACACCAGCAACCAAAGAAGAAGTCAAAGCTGCGATCCCAAACGGATCTGAAGGAAGGGACATAAAGCGAACACGCCCAGGGAAGCCCAGAGGAGAAACTCCCGGGCAACTGCTTCTGGAGATCATGCCAGAGGATGGAGTCTCGCGAGAGTCTGGTCAAAACCCTCGTGAGGCTCAAAGATCGGCTGAGGCACTCTTCAGGCTGCAGGCCATGGCCA

>Senegal Kedougou31 2017 (MW281023)

CCTTCCTCCAGCACAAAATAGGTGAGGGAGAGTCGCCTACACCAGCAACCAAAGAAGAAGTCAAAGCTGCGATCCCAAACGGATCTGAAGGAAGGGACATAAAGCGAACACGCCCAGGGAAGCCCAGGGGAGAAACTCCCGGGCAACTGCTTCTGGAGATCATGCCAGAGGATGGAGTCTCGCGAGAGTCTGGTCAAAACCCTCGTGAGGCTCAAAGATCGGCTGAGGCACTCTTCAGGCTGCAGGCCATGGCCA

>Senegal Kedougou14 2017 (MW281022)

CCTTCCTCCAGCACAAAACAGGTGAGGGAGAGTCGCCTACACCAGCGACCAAAGAAGAAGTCAAAGCTGCGATCCCAAACGGATCTGAAGGAAGAGACACAAAACGAACACGCCCAGGGAAGCCCAGAGGAGAAACTCCCGGGCAACTGCTTCTGGAGATCATGCCAGAGGATGGAGTCCCGCGAGAGTCTGGTCAAAACCCTCGTGAGGCTCAAAGATCGGCTGAGGCACTCTTCAGGCTGCAGGCCATGGCCA

>Senegal Tambacounda33 2017 (MW281020)

CCTTCCTCCAGCACAAAATAGGTGAGGGAGAGTCGCCTACACCAGCGACCAAAGAAGAAGTCAAAGCTGCGATCCCAAACGGATCTGAAGGAAGAGACACAAAACGAACACGCCCAGGGAAGCCCAGAGGAGAAACTCCCGGGCAACTGCTTCTGGAGATCATGCCAGAGGATGGAGTCTCGCGAGAGTCTGGTCAAAACCCTCGTGAGGCTCAAAGATCGGCTGAGGCACTTTTCAGGCTGCAGGCCATGGCCA

>Senegal Tambacounda21 2017 (MW281024)

CCTTCCTCCAGCACAAAATAGGTGAGGGAGAGTCGCCTCCACCAGCGACCAAAGAAGAAGTCAAAGCTGCGATCCCAAACGGATCTGAAGGAAGAGACACAAAACGAACACGCCCAGGGAAGCCCAGAGGAGAAACTCCCGGGCAACTGCTTCTGGAGATCATGCCAGAGGATGGAGTCTCGCGAGAGTCTGGTCAAAACCCTCGTGAGGCTCAAAGATCGGCTGAGGCACTTTTCAGGCTGCAGGCCATGGCCA

>Mali Dialan CP2 2015 (MW281010)

CTTTCCTCCAGCACAAAACAGTAGAGGGAGAGTCATCTGCACCAGTGACCAGAGAAGAAGTCAAGGCTGCGATTCCAAACGGGTCCGAAGAAAGGGACAAAAGGCGAACCCGCCCAGGAAGGCCCAGAGGAGAAACCCCGAGCCAACCGCTCCTGGAAATCATGTCAGAGGATGAGGCTTCGAGAGAATCCGGCCAAGCCTCTCGTGAGGCTCAGAGGTCGGCCGAGGCACTTTTCAGGCTGCAAGCTATGGCCA

>Mali Dialan CP1_2015 (MW281010)

CTTTCCTCCAGCACAAAACAGTAGAGGGAGAGTCATCTGCACCAGTGACCAGAGAAGAAGTCAAGGCTGCGATTCCAAACGGGTCCGAAGAAAGGGACAAAAGGCGAACCCGCCCAGGAAGGCCCAGAGGAGAAACCCCGAGCCAACCGCTCCTGGAAATCATGTCAGAGGATGAGGCTTCGAGAGAATCCGGCCAAGCCTCTCGTGAGGCTCAGAGGTCGGCCGAGGCACTTTTCAGGCTGCAAGCTATGGCCA

>Mali Segou 2F 2014 (MW281012)

CTTTCCTCCAGCACAAAACAGTAGAGGGAGAGTCATCTGCACCAGTGACCAGAGAAGAAGTCAAGGCTGCGATTCCAAACGGGTCCGAAGAAAGGGACAAAAGGCGAACCCGCCCAGGAAGGCCCAGAGGAGAAACCCCGAGCCAACCGCTCCTGGAAATCATGCCAGAGGATGGGGCTTCGCGAGAGTCCGGCCAAGCCCCTCGTGAGGCTCAGAGGTCGGCCGAGGCACTCTTCAGGCTGCAAGCTATGGCCA

>Mali Segou F9 2014 (MW281013)

CTTTCCTCCAGCACAAAACAGTAGAGGGAGAGTCATCTGCACCAGTGACCAGAGAAGAAGTCAAGGCTGCGATTCCAAACGGGTCCGAAGAAAGGGACAAAAGGCGAACCCGCCCAGGAAGGCCCAGAGGAGAAACCCCGAGCCAACCGCTCCTGGAAATCATGCCAGAGGATGAGGCTTCGAGAGAATCCGGCCAAGCCTCTCGTGAGGCTCAGAGGTCGGCCGAGGCACTCTTCAGGCTGCAAGCTATGGCCA

>Mali Segou F1 2014 (MW281014)

CTTTCCTCCAGCACAAAACAGTAGAGGGAGAGTCATCTGCACCAGTGACCAGAGAAGAAGTCAAGGCTGCGATTCCAAACGGGTCCGAAGAAAGGGACAAAAGGCGAACCCGCCCAGGAAGGCCCAGAGGAGAAACCCCGAGCCAACCGCTCCTGGAAATCATGCCAGAGGATGAGGCTTCGAGAGAATCCGGCCAAACCTCTCGTGAGGCTCAGAGGTCGGCCGAGGCACTTTTCAGGCTGCAAGCTATGGCCA

>Mali Segou M6 2014 (MW281015)

CTTTCCTCCAGCACAAAACAGTAGAGGGAGAGTCATCTGCACCAGTGACCAGAGAAGAAGTCAAGGCTGCGATTCCAAACGGGTCCGAAGAAAGGGACAAAAGGCGAACCCGCCCAGGAAGGCCCAGAGGAGAAACCCCGAGCCAACCGCTCCTGGAAATCATGCCAGAGGATGAGGCTTCGAGAGAATCCGGCCAAACCTCTCGTGAGGCTCAGAGGTCGGCCGAGGCACTTTTCAGGCTGCAAGCTATGGCCA

>Mali Segou 1F 2014 (MW281016)

CTTTCCTCCAGCACAAAACAGTAGAGGGAGAGTCATCTGCACCAGTGACCAGAGAAGAAGTCAAGGCTGCGATTCCAAACGGGTCCGAAGAAAGGGACAAAAGGCGAACCCGCCCAGGAAGGCCCAGAGGAGAAACCCCGAGCCAACCGCTCCTGGAAATCATGCCAGAGGATGAAGTTTCGAGAGAATCCGGCCAAACCCCTCGTGAGGCTCAGAGGTCGGCCGAGGCACTCTTCAGGCTGCAAGCTATGGCCA

>Algeria 2012(KP793696)

CCTTCCTCCAGCACAAAACAGGAGAGGGAGATTCGCCCGCACCAGCGACCAGAGAAGGGGTCAAAGCTGCGATCTCAAACGGATCTGAAGAAAGGGATAGAAAGCATACACGCCCAGGAAGGCCCAGAGGAGAGACCCCCGGTCAACTGCTCCTGGAAATCATGCCAGAGGACGAGGCTTCGCGAGAGTCTGGTCAAAACCCTCGTGAGGCTCAAAGATCAGCCGAGGCACTCTTCAGGCTGCAGGCTATGGCCA

>Burkina Faso Pibaore 2014 (MK777903)

CCTTCCTCCAGCACAAAATAGGTGAGGGAGAGTCGCCTACACCAGCAACCAAAGAAGAAGTCAAAGCTGCGATCCCAAACGGATCTGAAGGAAGGGACACAAAGCGAACACGCCCAGGGAAGCCCAGAGGAGAAACTCCCGGGCAACTGCTTCTGGAGATCATGCCAGAGGATGGAGTCTCGCGAGAGTCGGGTCAAAACCCTCGTGAGGCTCAAAGATCGGCTGAGGCACTCTTCAGGCTGCAGGCCATGGCCA

>Burkina Faso Binde 2014 (MK777897)

CCTTCCTCCAGCACAAAATAGGTGAGGGAGAGTCGCCTACACCAGCGACCAAAGAAGAAGTCAAAGCTGCGATCCCAAACGGGTCCGAAGGAAGGGACATAAAGCGAACACGCCCAGGGAAGCCCAGAGGAGAAACTCCCGGCCAACTGCTTCTGGATATCATGCCAGAGGATGAAGTCTCGCGAGAGTCTGGTCAAAACCCTCGTGAGGCTCAAAGATCGGCTGAGGCACTTTTCAGGCTGCAGGCCATGGCCA

>Ghana 2014 (MK777904)

CCTTCCTCCAGCACAAAATAGGTGAGGGAGAGTCGCCTACACCAGCGACCAAAGAAGAAGTCAAAGCTGCGATCCCAAACGGGTCCGAAGGAAGGGACATAAAGCGAACACGCCCAGGGAAGCCCAGAGGAGAAACTCCCGGCCAACTGCTTCTGGAGATCATGCCAGAGGATGAAGTCTCGCGAGAGTCTGGTCAAAACCCTCGTGAGGCTCAAAGATCGGCTGAGGCACTTTTTAGGCTGCAGGCCATGGCCA

>Benin 2011(KT692542)

CCTTCCTCCAGCACAAAATGGGTGAGGGAGAGTCGCCTACACCAGCGACCAAAGAAGAAGTCAAAGCTGCGATCCCAAACGGGTCCGAAGGAAGGGACATAAAGCGAACACGCCCAGGGAAGCCCAGAGGAGAAACTCCCGGCCAACTGCTTCTGGAGATCATGCCAGAGGATGAAGTCTCGCGAGAGTCTGGTCAAAACCCTCGTGAGGCTCAAAGAT---------------------

---------------

>Niger 2016 (MK673127)

CCTTCCTCCAGCACAAAATAGGTGAGGGAGAGTCGCCCACACCAGCAACCAAAGAAGAAGTCAAAGCTGCGATCCCAAACGGATCTGAAGGAAGGGACATAAAGCGAACACGCCCAGGGAAGCCCAGAGGAGAAACTCCCGGGCAACTGCTTCTGGAGATCATGCCAGAGGATGGAGTCTCGCGAGAGTCTGGTCAAAACCCTCGTGAGGCTCAAAGAT------------------------------------

>Benin 2011(KT692538)

CCTTCCTCCAGCACAAAATAGGTGAGGGAGAGTCGCCTACACCAGCGACCAAAGAAGAAGTCAAAGCTGCGATCCCAAACGGATCCGAAGGAAGAGACATAAAGCAAACACGCCCAGGGAAGCCCAGAGGAGAAACTCCCGGGCAACTGCTTCTGGAGATCATGCCAGAGGATGGAGTCTCGCGAGAGTCTGGTCAAAACCCTCGTGAGGCTCAAAGAT------------------------------------

>Gambia 2012 (MG581408)

CCTTCCTCCAGCACAAAATAGGTGAGGGAGAGTCGCCTACACCAGCGACCAAAGAAGAAGTCAAAGCTGCGATCCCAAACGGATCCGAAGGAAGAGACATAAAGCAAACACGCCCAGGGAAGCCCAGAGGAGAAACTCCCGGGCAACTGCTTCTGGAGATCATGCCAGAGGATGGAGTCTCGCGAGAGTCTGGTCAAAACCCTCGTGAGGCTCAAAGAT------------------------------------

>Mali Tousseguela 2014 (MK777892)

CCTTCCTCCAGCACAAAACAGGCGAGGGAGAGTCGCCTACACCAGCGACCAAAGAAGGAGTCAAAGCTGCGATCCCAAACGGATCCGAAGGAAGGGACATAAAGCGAACACGCCCAGGGAAGCCCAGAGGAGAAACTCCCGGGCAACTGCTTCTGGAGATCATGCCAGAGGATGAATTCTCGCGAGAGTCTGGTCAAAACCCTCGTGAGGCTCAAAGATCGGCTGAGGCACTCTTCAGGCTGCAGGCCATGGCCA

>Mali Samako 2014 (MK777889)

CCTTCCTCCAGCACAAAACAGGCGAGGGAGAGTCGCCTACACCAGCGACCAAAGAAGAAGTCAAAGCTGCGATCCCAAACGGATCCGAAGGAAGGGACATAAAGCGAACACGCCCAGGGAAGCCCAGAGGAGAAACTCCCGGGCAACTGCTTCTGGAGATCATGCCAGAGGATGAATTCTCGCGAGAGTCTGGTCAAAACCCTCGTGAGGCTCAAAGATCGGCTGAGGCACTCTTCAGGCTGCAGGCCATGGCCA

>Niger Falmey 2001 (MG694471)

CCTTCCTCCAGCACAAAACAGGTGAGGGAGAGTCGCCTACACCAGCGACCAAAGAAGAAGTCAAAGCTGCGATCCCAAACGGATCCGAAGGAAGGGACATAAAGCGAACACGCCCAGGGAAGCCCAGAGGAGAAACTCCCGGCCAACTGCTTCTGGAGATCATGCCAGAGGATGAAGTCTCGCGAGAGTCTGGTCAAAACCCTCGTGAGGCTCAAAGATCGGCTGAGGCACTCCTCAGGCTGCAGGCCATGGCCA

>Ghana 2010 (KJ676599)

CCTTCCTCCAGCACAAAATTGGTGAGGGAGAGTCGCCTACACCAGCGACCAAAGAAGAAGTTAAAGCTGCGATCCCAAACGGATCTGAAGGAAGGGACATAAAGCGAACACGCCCAAGGAAGCCCAGAGGAGAAACTCCCGGGCAACTGCTTCTGGAGATCATGCCAGAGGATGAAGCCTCGCGAGAGTCTGGTCAAAACCCTCGTGAGGCTCAAAGATCGGCTGAGGCACTCTTCAGGCTGCAGGCCATGGCCA

>Nigeria Anambra 2010(KJ124765)

CCTTCCTCCAGCACAAACCGGGAGAGGGAGAGTCGCCCACACCAGCGACTAGAGAAGGGGTCAAAGCTGCGATCCCAAACGGATCTGAAGAGAGGGAAAGAAAGCAAACACGTTCAGGAAGGCCCAGAGGGGAGACCCCCGGCCAATTGCTCCTGGAAATCACGCCAGAGGATGAGGTCTCGCGAGAGTCTGGTCAAAACCCTCGTGAGGCTCAAAGATCGGCCGAGGCACTCTTCAGGCTGCAGGCCATGGCCA

>Burkina Faso Zegued 2008 (MK777898)

CCTTCCTCCAGCACAAAATAGGTGAGGGAGAGTCGCCTACACCAGCGACCAAAGAAGAAGTTAAAGCTGCGATCCCAAACGGATCTGAAGGAAGGGACATAAAGCGAACACGCCCAGGGAAGCCCAGAGGAGAAACTCCCGGGCAACTGCTTCTGGAGATCATGCCAGAGGATGAAGTCTCGCGAGAGTCTGGTCAAAACTCTCGTGAGGCTCAAAGATCGGCTGAGGCACTCTTCAGGCTGCAGGCCATGGCCA

>BurkinaFasso 1988(DQ840172)

CTTTCCTCCAGCATAAAACAGATGAGGGAGAGTCATCTGCACCAGTGACCAGAGAAGAAGTCAAGGCTGCGATTCCAAATGGGTCCGAAGAAAGGGACAAAAGGCGAACCCGCCCAGGAAGGCCCAGAGGAGAAACCCCGAGCCAACCGCTCCTGGAAATCATGCCAGAGGATGAGGCCTCGAGAGAATCCGGCCAAACCTCTCGTGAGGCTCAGAGGTCGGCCGAGGCACTCTTCAGGCTGCAAGCTATGGCCA

>Comores 2013(KM669158)

CCTTCCTCCAGCATAAAATAGGAGAGGGAGAGTCACATGCATCGGCGACCAGGGAAGAAGTCAAAGCTGCGACCCCAAATGGGCCCGACGAAAAGGACAAAACTCGGGCGCGCTCAGGAAAGCCAAGAGGAGGAACCCCCGACCAACTGCTCCTGGAAATCATGCCTGAAGACGAGGTCCCGCGAGGGTCTGGACAAAACCCTCGTGAGGCTCAAAGATCGGCCGAGGCACTCTTTAGACTGCAGGCCATGGCCA

>Dubai 2009(FJ795511)

CCTTCCTTCAGCACCAAACAGGAGGGGGAGAGTCGTCCGCACCAGCGACCAGAGAAGGGGTCAAATCTGCGATCCCAAACGGATCTGAAGAAAGGGACAGAAAGCAAACACGCCCAGGAAGGCCCAGAGGAGAGACCTCCGGCCAACTGCTCCTGGACATCATGCCAGAGGATGAGGTCTCGCGAGAGTCTGGTCAAAACCCTCGTGAGGCTCAAAGATCGGCCGAGGCACTCTTCAGGCTGCAGGCCATGGCCA

>Ethiopia 1996(DQ840183)

CCTTCCTCCAGCACAAAATAGGAGAGGGAGAGTCACATGCATCGGCGACCAGGGAAGAAGTCAAAGCTGCGACCCCACATGGGCCCGACGAAAAGGGCAAAACTCGGGCACGCTCAGGAAGGCCAAGAGGAGGAACCCCCGACCAACTGCTCTTGGAAATCATGCCTGAAGACGAGGTCCCGCGAGGGTCTGGACAAAACCCTCGTGAGGCTCAAAGATCGGCCGAGGCACTCTTTAGACTGCAGGCCATGGCCA

>Ghana 1978(DQ840166)

CCTTCCTCCAGCACAAAATAGATGAGGGAGATGCGCCTACACCAGCGACCAGAGAAGAAGTCAAGGCTGCGATCCCAAATGGGTCCGAAGGAAGGGAGCCAAAGCGAACACGCTCAGGAAAGCCCAGAGGAGAAACTCCCGGCCAACTGCTTCTGGAGATCATGCCAGAGGATGAAGTCTCGCGAGAGTCTAGTCAAAACCCTCGTGAGGCTCAAAGATCGGCTGAGGCACTCTTCAGGCTGCAGGCCATGGCCA

>Guinea 1988(DQ840170)

CTTTCCTCCAGCATAAAACGGGAGAGGGAGAGTCATCTGCACCAGTGACCAGAGAAGAAGTCAAGACTGCGATTCCAAATGGGTCCGAAGAAAGGGACAAAAGGCGAGCCCGCTCAGGAAGGCCCAGAGGAGAAACCCCGAGCCAACCGCTCCTGGAAATCATGCCAGAGGATGAGGCCTCGGGGGAATCCGGTCAAACCCCTCGTGAGGCTCAGAGGTCGGCAGAGGCACTCTTCAGGCTGCAAGCTATGGCCA

>Ivory Coast 2009 (KR781451)

CCTTCCTCCAGCACAAAATAGGTGAGGGAGAGTCGCCTACACCAGCAACCAAAGAAGAAGTCAAAGCTGCGATCCCAAACGGATCTGAAGGAAGGGACATAAAGCGAACACGCCCAGGGAAGCCCAGAGGAGAAACTCCCGGGCAACTGCTTCTGGAGATCATGCCAGAGGATGGAGTCTCGCGAGAGTCTGGTCAAAACCCTCGTGAGGCTCAAAGATCGGCTGAGGCACTTTTCAGGCTGCAGGCCATGGCCA

>Mali 1999(DQ840192)

CCTTCCTCCAGCGCAAAATAGGTGAGGGAGAGTCGCCTACACCAGCGACCAAAGAAGAAGTCAAAGCTGCGATCCCAAACGGGTCTGAAGGAAGGGACATGAAGCGAACACGCCCAGGGAAGCCCAGAGGAGAAACTCCCGGCCAACTGCTTCTGGAGATCATGCCAGAGGATGAAGTCTCGCGAGAGTCTGGTCAAAACCCTCGTGAGGCTCAAAGATCGGCTGAGGCACTCTTCAGGCTGCAGGCCATGGCCA

>Senegal Dakar5 2013 (MK777907)

CCTTCCTCCAGCACAAAATAGGTGAGGGAGAGTCGCCTACACCAGCGACCAAAGAAGAAGTCAAAGCTGCGATCCCAAACGGATCCGAAGGAAGAGACATAAAGCGAACACGCCCAGGGAAGCCCAGAGGAGAAACTCCCGGGCAACTGCTTCTGGAGATCATGCCAGAGGATGGAGTCTCGCGAGAGTCTGGTCAAAACCCTCGTGAGGCTCAAAGATCGGCTGAGGCACTCTTCAGGCTGCAGGCCATGGCCA

>Mauritania 2012(KF483658)

CCTTCCTCCAGCACAAAATAGGTGAGGGAGAGTCGCCTACACCAGCGACCAAAGAAGAAGTCAAAGCTGCGATCCCAAACGGATCTGAAGGAAGAGACATAAAGCGAACACGCCCAGGGAAGCCCAGAGGAGAAACTCCCGGGCAACTGCTTCTGGAGATCATGCCAGAGGATGGAGTTTCGCGAGAGTCTGGTCAAAACTCTCGTGAGGCTCAAAGATCGGCTGAGGCACTCTTCAGGCTGCAGGCCATGGCCA

>Mauritania 2014 (MK777900)

CCTTCCTCCAGCACAAAATAGGTGAGGGAGAGTCGCCTACACCAGCGACCAAAGAAGAAGTCAAAGCTGCGATCCCAAACGGATCTGAAGGAAGAGACACAAAACGAACACGCCCAGGGAAGCCCAGAGGAGAAACTCCCGGGCAACTGCTTCTGGAGATCATGCCAGAGGATGGAGTCTCGCGAGAGTCTGGTCAAAACCCTCGTGAGGCTCAAAGATCGGCTGAGGCACTCTTCAGGCTGCAGGCCATGGCCA

>Niger 2013(MG694464)

CCTTCCTCCAGCACAAACCGGGAGAGGGAGAGTCGCCCACACCAGCGACCAGAGAAGGGGTCAAAGCTGCGATCTCAAACGGATCTGAAGAGAGGGAAAGAAAGCAAGCACGCTCAGGAAGGCCTAGAGGGGAGACCCCTGGCCAACTGCTCCTGGAAATTATGCCAGAGGATGAGGTCTCGCGAGAGTCTGGTCAAAACCCTCGTGAAGCTCAAAGATCGGCCGAGGCACTCTTCAGGCTGCAGGCCATGGCTA

>Niger 2001(MG694466)

CCTTCCTCCAGCACAAAACAGGTGAGGGAGAGTCGCCTACACCAGCGACCAAAGAAGAAGTCAAAGCTGCGATCCCAAACGGATCCGAAGGAAGGGACATAAAGCGAACACGCCCAGGGAAGCCCAGAGGAGAAACTCCCGGCCAACTGCTTCTGGAGATCATGCCAGAGGATGAAGTCTCGCGAGAGTCTGGTCAAAACCCTCGTGAGGCTCAAAGATCGGCTGAGGCACTCCTCAGGCTGCAGGCCATGGCCA

>Nigeria 1976(DQ840164)

CCTTCCTCCAGCATAAAATAGATGAGGGAGAGTCGCCTACACCAGCGACCAGAGAAGAATTCAAAGCTACGATCCCAAATGGGTCTGAAGGAAGGGACACAAAGCGAACACGCTCAGGAAAACCCAGAGGAGAAACTCCCGGCCAACTGCTTCTGGAGATCATGCCAGAGGATGAAGTCTCGCGAGAGTCTAGTCAAAACCCTCGTGAGGCTCAAAGATCGGCTGAGGCACTCTTCAGGCTGCAGGCCATGGCCA

>Niger 2001(MG694468)

CTTTCCTCCAGCACAAAACAGTAGAGGGAGAGTCATCTGCACCAGTGACCAGAGAAGAAGTCAAGGCTGCGATTCCAAACGGGTCCGAAGAAAGGGACAAAAGGCGAACCCGCCCAGGAAGGCCCAGAGGAGAAACCCCGAGCCAACCGCTCCTGGAAATCATGCCAGAGGATGAGGCTTCGAGAGAATCCGGCCAAACCTCTCGTGAGGCTCAGAGGTCGGCCGAGGCACTTTTCAGGCTGCAAGCTATGGCCA

>Oman 1983(DQ840168)

CCTTCCTCCAGCATAAAACAGGAGAGGGAGAGTCACATGCATCGGTGACCAGGGAAGAAGTCACAGCTGAGACCCCAAATGGGCCCGACGAGAAGGACAAGAAACGAGCACGCCCAGGAAGGCCAAGAGGAGGAACCCCCGACCAACTGCTCCTGGAGATCATGCCTGAAGACGAGGTCCCGCGAGGGCCTGGACAAACCCCTCGTGAGGCTCAACGATCGGCGGAGGCACTCTTTAGACTACAGGCCATGGCCA

>Senegal 1968(DQ840165)

CTTTCCTCCAGCACAAAACAGGAGTGGGAGAGTCATCTGCACCAGTGACCAGAGAAGAAGTCAAGGCTGCGATTCCAAATGGGTCCGAAGAAAGGGACAAAAGGCGAACCCGCTCAGGAAGACCCAGAGGAGAGACCCCGGGCCAACCGCTCTTGGAAATCATGCCAGAGGATGAGGTCTCGCGAGAATCTGGCCAAACCCCTCGTGAGGCTCAGAGGTCGGCCGAGGCACTCCTCAGGCTGCAAGCCATGGCCA

>Senegal 1994(DQ840174)

CTTTCCTCCAGCATAAAACAGTAGAGGGAGAGTCATCTGCACCAGTGACCAGAGAAGAAGTCAAGGCTGCGATTCCAAATGGGTCCGAAGAAAGGGACAAAAGGCGAACCCGCCCAGGAAGGCCCAGAGGAGAAACCCCGAGCCAACCGCTCCTGGAAATCATGCCAGAGGATGGGGCCTCGAGAGAATCCGGCCAAACCTCTCGTGAGGCTCAGAGGTCGGCCGAGGCACTCTTCAGGCTGCAAGCTATGGCCA

>India 2016(KX033350)

CCTTCCTACAGCACCAAACAGGAGGGGGAGAGTCGTCCGCACCAGCGACCAGAGAAGGGGTCAAAGCTGCGATCCCAAACGGATCCGAAGAAAGGGACAGAAAGCAAACACGCCCAGGGAGGCCCAGAGGAGAGACCCCCGGCCAACTGCTCCTGGAATTCATGCCAGAGGATGAGGTCTCGCGAGAATCTGGTCAAAACCCTCGTGAGGCTCAAAGGTCGGCCGAGGCACTCTTCAGGCTGCAGGCCATGGCCA

>Egypt 2015(KX189064)

CCTTCCTCCAGCACAAAGCAGGAGAGGGAGATTCGCCCGCACCAGCCACCAGAGAAGGGGTCAAAGCTGCGATCTCAAACGGATCTGAAGAAAGGGACAGAAGGCAAACACGCCCAGGAAGGCCCAGAGGAGAGACCCCCGGTCAACTGCTCCTGGAAATCATGCCAGAGGATGAGGTCTCGCGAGAGTCTGGTCAAAACCCTCGTGAGGCTCAAAGATCAGCCGAGGCACTCTTCAGGCTGCAGGCCATGGCCA

>Morocco 2015(KY197740)

CCTTCCTCCAGCACAAAACAGGAGAGGGAAATTCGCCCGCACCAGCGACCAGAGAAGGGGTCAAAGCTGCAATCTCAAACGGATCTGAAGAAAGGGATAGAAGCCAGGCACGCCCAGGAAGGCCCAGAGGAGAGACCCCCGGTCAACTGCTCCTGGAAATCATGCCAGAGGACGAGACTTCGCGAGAGTCTGGTCAAAACCCTCGTGAGGCTCAAAGATCAGCCGAGGCACTCTTCAGGCTGCAGGCTATGGCCA

>Ethiopia 2014(KX816961)

CCTTCCTCCAGCACAAAGCAGGAGAGGGGGATTCGCCCGCACCAGCGACCAGAGAAGGGGTCAAAGCTGCGGTCTCGAACGGATCTGAAGAAAGGGACAGAAAGCAAACACGCCCAGGAAGGCCCAGAGGAGAGACCCCCGGTCAAATGCTCCTGGAAATCATGCCAGAGGATGAGGTCTCGCGAGAGTCTGGTCAAAACCCTCGTGAGGCTCAAAGATCAGCCGAGGCACTCTTCAGGCTGCAGGCCATGGCCA

>Sierra Leone 2009(JN602080)

CCTTCCTCCAGCACAAAATAGGTGAGGGAGAGTCGCCTACACCAGCGACCAAAGAAGAAGTCAAAGCTGTGATCCCAAACGGATCTGAAGGAAGAGACATAAAGCGAACACGCCCAGGGAAGCCCAGAGGAGAAACTCCCGGGCAACTGCTTCTGGAGATCATGCCAGAGGATGGAGTCTCGCGAGAGTCTGGTCAAAACCCTCGTGAGGCTCAAAGATCGGCTGAGGCACTCTTCAGGCTGCAGGCCATGGCCA

>Gabon 2011(JX079996)

CCTTCCTCCAGCACAAACCGGGAGAGGGAGAGTCGCCCACACCAGCGACCAGAGAAGGGGTCAAAGCTGCAATCTCAAACGGATCTGAAGAGAGGGAAAGAAAGCAAACACGCTCAGGAAGGCCTAGAGGGGAGACCCCTGGCCAACTGCTCCTGGAAATCATGCCAGAGGATGAGGTCTCGCGAGAGTCTGGTCAAAACCCTCGTGAGGCTCAGAGATCGGCCGAGGCACTCTTCAGGCTGCAGGCCATGGCCA

>Cameroon 2017 (MH447978)

CCTTCCTCCAGCACAAACCGGGAGAGGGAGAGTCGCCCACACCAGCGACCAGAGAAGGGGTCAAAGCTGCGATCCCAAACGGATCTGAAGAGAGGGAAAGAAAGCAAACACGCTCAGGAAGGCCCAGAGGGGAGACCCCCGGCCAACTGCTCCTGGAAATCACGCCAGAGGATGAGGTCTCGCGAGAGTCTGGTCAAAACCCTCGTGAGGCCCAAAGATCGGCCGAGGCACTCTTCAGGCTGCAGGCCATGGCCA

>Angola 2012 (KP189203)

CCTTCCTCCAGCACAAACCGGGAGAGGGAGAGTCGCCCACACCAGCGACCAGAGAAGGGGTCAAAGCTGCGATCTCAAACGGATCTGAAGAGAGGGAAAGAAAGCAAACACGCTCAGGAAGGCCCAGAGGGGAGACCCCTGGCCAACTGCTCCTGGAAATCATGCCAGAGGATGAGGTCTCGCGAGAGTCTGGTCAAAACCCTCGTGAGGCTCAAAGATCGGCCGAGGCACTCTTCAGGCTACAGGCCATGGCCA

>Niger 2017 (MK673129)

CCTTCCTCCAGCACAAACCGGGAGAGGGAGAGTCGCCCACACCAGCGACCAGAGAAGGGGTCAAAGCTGCGATCCCAAACGGATCTGAAGAGAGGGAAAGAAAGCAAACACGCTCAGGAAGGCCCAGAGGGGAGACCCCTGGACAATTGCTCCTGGAAATCACGCCAGAGGATGAGGTCCCGCGAGAATCTGGTCAAAACCCTCGTGAGGCTCAAAGATCGGCCGAGGCACTCTTCAGGCTACAGGCCATGGCCA

>Soudan 2008(HQ131921)

CCTTCCTCCAGCACAAACCGGGAGAGGGAGAGTCGCCCACACCAGCGACCAGAGAAGGGGTCAAAGCTGCGATCCCAAACGGATCTGAAGAGAGGGAAAGAAAGCAAACACGCTCAGGAAGGCCCAGAGGGGAGACCCCCGGCCAACTGCTCCTGGAAATCATGCCAGAGGATGAGGTCTCGCGAGAGTCTGGTCAAAACCCTCGTGAGGCTCAAAGATCGGCCGAGGCACTCTTCAGGCTGCAGGCTATGGCCA

>China 2014(KP260624)

CCTTCCTTCAGCACAAAACAGGAGAGGGAGAGTCGTCCGCACCAGCAACCAGAGAGGGGGTCAAAGCTGCGATCCCAAACGGAGCCGAAGAAAGGGACAGGAAGCAAACACGCTCAGGAAGGCCCAGAGGAGAGACCTCCGGCCAACTGCTCCTGGACATCATGCCAGAGGATGAGATCTTGCGAGAGTCTGGTCAAAACCCTCGTGAGGCTCAAAGATCGGCCGAGGCACTCTTCAGGCTGCAGGCCATGGCCA

>Senegal 2010(HQ131963)

CCTTCCTCCAGCACAAAATAGGTGAGGGAGAGTCGCCTACACCAGCGACCAAAGAAGAAGTCAAAGCTGCGATCCCAAACGGATCTGAAGGAAGAGACATAAAGCGAACACGCCCAGGGAAGCCCAGAGGAGAAACTCCCGGGCAACTGCTTCTGGAGATCATGCCAGAGGATGGAGTCTCGCGAGAGTCTGGTCAAAACCCTCGTGAGGCTCAAAGATCGGCTGAGGCACTCTTCAGGCTGCAGGCCATGGCCA

>Nigeria 2013(KF908036)

-----------CACAAAATAGGTGAGGGAGAGTCGCCTACACCAGCGACCAAAGAAGAAG

TCAAAGCTGCGATTCCGAACGGGTCCGAAGGAAGGGACATAAAGCGAACACGCCCAGGGAAGCCCAGAGGAGAAACTCCCGGCCAACTGCTTCTGGAGATCATGCCAGAGGATGAAGTCTCGCGAGAGTCTGGTCAAAACCCTCGTGAGGCTCAAAGATCGGCTGAGGCACTTTTCAGGCTGCAGGCCATGGCCA

>Nigeria 2013(KF908047)

-----------CACAAACCGGGAGAGGGAGAGTCGCCCACACCAGCGACCAGAGAAGGGG

TCAAAGCTGCGATCCCAAACGGATCTGAAGAGAGAGAAAGAAAGCAAACACGCTCAGGAAGGCCCAGAGGGGAGACCCCCGGCCAATTGCTCCTGGAAATCACGCCAGAGGATGAGGTCTTGCGAGAGTCTGGTCAAAACCCTCGTGAGGCTCAAAGATCGGCCGAGGCACTCTTCAGGCTGCAGGCCATGGCCA

>Nigeria 2012(KJ124730)

CCTTCCTCCAGCACAGACCGGGAGAGGGAGAGTCGCCCACACCAGCGACCAGAGAAGGGGTCAAAGCTGCGATCTCAAACGGATCTGAAGAGAGGGAAAGAAAGCAAACACGCTCAGGAAGGCCTAGAGGGGAGACCCCTGGCCAACTGCTCCTGGAAATCATGCCAGAGGATGAAGTCTCGCGAGAGTCTGGTCAAAACCCTCGGGAGGCTCAAAGATCGGCCGAGGCACTCTTCAGGCTGCAGGCCATGGCCA

>Ghana 2010(KJ676597)

CCTTCCTCCAGCACAAAATTGGTGAGGGAGAGTCGCCTACACCAGCGACCAAAGAAGAAGTTAAAGCTGCGATCCCAAACGGATCTGAAGGAAGGGACATAAAGCGAACACGCCCAAGGAAGCCCAGAGGAGAAACTCCCGGGCAACTGCTTCTGGAGATCATGCCAGAGGATGAAGCCTCGCGAGAGTCTGGTCAAAACCCTCGTGAGGCTCAAAGATCGGCTGAGGCACTCT------

---------------

>Turkey 2010(JQ388660)

CCTTCCTCCAGCACAAAACAGGAGAGGGAGAGTCGTCCGCACCAGCAACCAGAGAAGGGGTCAAAGCTGCGATCCCAAACGGATCCGAAGAAAGGGACAGAAAGCAAACACGCCCAGGAAGGTCCAGAGGAGAGACCCCCAGCCAACTGCTCCTGGAAATCATGCCAGAGGATGAGGTCTCGCGAGAGTCTGGTCAAAACCCTCGTGAGGCTCAAAGATCGGCCGAGGCACTCTTCAGGCTGCAGGCCATGGCCA
